# Simulation of future COVID-19 epidemic by vaccination coverage scenarios in Japan

**DOI:** 10.7189/jogh.11.05025

**Published:** 2021-11-30

**Authors:** Yuki Furuse

**Affiliations:** 1 Institute for Frontier Life and Medical Sciences, Kyoto University, Kyoto, Japan; 2 Hakubi Center for Advanced Research, Kyoto University, Kyoto, Japan; 3 Nagasaki University Graduate School of Biomedical Sciences, Nagasaki, Japan

## Abstract

**Background:**

COVID-19 continues to impose significant morbidity and mortality in Japan even after implementing the vaccination program. It would remain elusive if restrictions for its mitigation were to be lifted or relaxed in the future.

**Methods:**

A simulation study that explored possible vaccination coverage scenarios and changes in the intensity of nonpharmaceutical intervention restrictions was performed to assess the impact of COVID-19 based on death count.

**Results:**

Assuming the basic reproduction number of circulating viruses was 5.0, vaccines could prevent 90% of infections and 95% of deaths, and the vaccination coverage rate was high (75%, 80%, and 90% in people aged 12-39 years, 40-59 years, ≥60 years, respectively), approximately 50 000 deaths would occur over 150 days in Japan if all restrictions were lifted. Most deaths would occur among older adults, even if their vaccination coverage was assumed to be especially high. A low vaccination coverage scenario (45%, 60%, and 80% in people aged 12-39 years, 40-59 years, ≥60 years, respectively) would require periodic implementation of strict measures even if the modified lifestyle observed in 2020 was sustained and vaccines were very effective. Some restrictions could be relaxed under high vaccination coverage. However, in the worst-case scenario where vaccines had decreased efficacy, as we have observed for the Delta variant, and people lived a relaxed lifestyle, our simulation suggests that even high vaccination coverage would occasionally require strict measures.

**Conclusions:**

We should carefully explore a manageable degree of restrictions and their relaxation. We will have to keep bracing for occasional surges of COVID-19 infection, which could lead to strict measures, such as those under a state of emergency. Such strategies are essential even after a wide rollout of vaccination.

COVID-19 caused a pandemic in 2020 and has affected many countries, including Japan. The first COVID-19 case in the country was confirmed on 15 January 2020 [1], and the cumulative numbers of cases and deaths reached 1 million ( ~ 8 per 1000 population) and 15 000 ( ~ 1 per 10 000 population), respectively, in August 2021. Nonpharmaceutical interventions (NPIs), such as physical distancing, wearing a face mask, rapid case detection, contact tracing, and isolation, play a significant role in controlling the COVID-19 epidemic [2,3]. When COVID-19 cases surged and concern about the collapse of health systems grew in Japan, a state of emergency was issued—asking people to stay at home and limit mass gatherings and asking businesses, including restaurants and bars, to reduce their hours or close. Although these measures were not mandatory but rather advisory, many citizens voluntarily followed them. As a result, the number of cases eventually decreased [4].

Several vaccines for COVID-19 were developed and became in use within one year after the emergence of SARS-CoV-2. In Japan, two vaccines, namely, BNT162b2 and mRNA-1273, were approved and administered to people ≥12 years old. These vaccines reportedly prevent ~ 90% of infections and ~ 95% of hospitalizations, severe illnesses, and deaths due to COVID-19 [5-10]. In Japan, vaccines were first administered to health care workers and those aged ≥65 years and then to other populations. The vaccination coverage for the prioritized groups reached ~ 80%-90%, and the number of COVID-19 cases among them dramatically dropped [11]. However, the vaccination coverage for the total population in Japan was still less than 50% as of August 2021 [12].

The Delta variant of SARS-CoV-2, which seems to have high transmissibility, was introduced and disseminated throughout Japan in June-August 2021, generating the fifth wave of the COVID-19 epidemic in the country [13]. While the basic reproduction number of the SARS-CoV-2 original strain was ~ 2.5 [14,15], the reproduction number of the Delta variant was estimated to be 5.0 [16]. In addition, the Delta variant reportedly decreases the efficacy of vaccines. Yet, vaccines still effectively prevent ~ 70%-90% of hospitalizations and fatal outcomes [17-20]. The pathogenicity of the Delta variant might have increased as well [21,22].

Whether a wide rollout of vaccination will control the spread of COVID-19 at a population level remains unknown. We thus performed a simulation study to assess the effect of vaccination on the impact of the disease by exploring several vaccination coverage scenarios in Japan. We also analyzed if and how NPI restrictions could be lifted or relaxed in the future.

## METHODS

**Figure 1:**
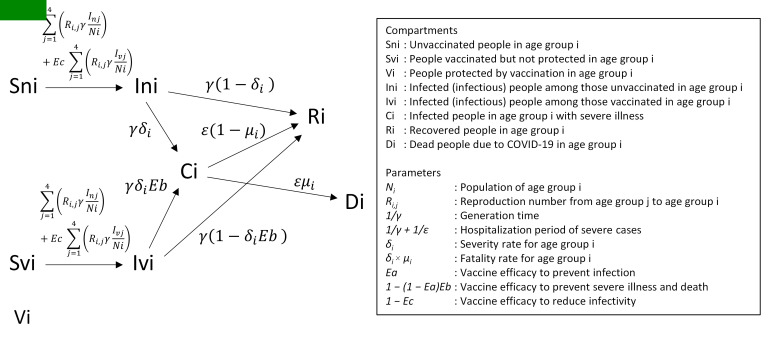
Compartmental model for the simulation. The compartmental model for age group *i* is shown. The model consists of four age groups. Arrows and italic characters respectively depict transitions between compartments and their rates. The compartments and parameters in the model are explained in Table S1 in the **Online Supplementary Document****.**

The spread of infection was simulated using a deterministic compartmental model with the following compartments: *S_n_*, susceptible, unvaccinated; *S_v_*, susceptible, vaccinated; *I_n_*, infectious, unvaccinated; *I_v_*, infectious, vaccinated; *C*, severe illness; *D*, death; *R*, recovered; and *V*, protected, vaccinated. The compartments were stratified into four age groups: 0-19, 20-39, 40-59, and ≥60 years. The details of the model and parameters are described in **Figure 1** and Table S1 in the **Online Supplementary Document**; the parameter values were based on empirical data from Japan.

The reproduction number of circulating viruses and the efficacy of vaccines were subject to change, and five scenarios of vaccination coverage were tested in the simulation (**Table 1**). The assumptions for these vaccination coverage scenarios were based on a July 2021 survey in Japan [23]. It was assumed that vaccination could decrease the probability of infection, severe illness, and death [5-10,17-20], and it also could reduce infectivity from patients with a breakthrough infection [24-26].

**Table 1:** Scenarios for the simulation*

**Basic reproduction number scenario**	**Value**	**Description**
	2.5	Corresponding to the original strain
	3.5	Corresponding to the Alpha variant
	5.0	Corresponding to the Delta variant
	7.5	Corresponding to 50% higher transmissibility than the current estimation for the Delta variant
**Vaccination coverage scenario**	**Vaccination coverage (12-39, 40-59, ≥60 years)**	**Description**
90% coverage	90%, 90%, 90%	Assuming 90% of eligible people got vaccinated
High coverage	75%, 80%, 90%	Corresponding to the sum of people who are willing to be vaccinated and those who cannot decide yet
Intermediate coverage	60%, 70%, 85%	Intermediate scenario between High and Low coverages
Low coverage	45%, 60%, 80%	Corresponding to a proportion of people who are willing to be vaccinated
No vaccination	0%, 0%, 0%	Scenario without vaccination
**Vaccine efficacy scenario**	**Efficacy to prevent infection, severe illness, death, and to decrease infectivity from patients with a breakthrough infection**	**Description**
Very effective	90%, 95%, 95%, 50%	Data from clinical trials and real-world data for the original strain and the Alpha variant
Effective	70%, 90%, 90%, 25%	Data for the Delta variant (the evidence is not yet sufficient)
**Transmission pattern scenario**		**Description**
Heterogeneous		Transmissions from people aged 20-59 years and transmissions within the same age group are higher than the other transmission pairs
Homogeneous		Transmission frequencies are the same among age groups

*Details of the scenarios and information sources are described in Table S1 in the **Online Supplementary Document.**

The effectiveness of past NPIs in Japan was calculated from time-dependent effective reproduction numbers of COVID-19 (Figure S1 in the **Online Supplementary Document**). A 40% reduction in transmission owing to NPIs was observed as a baseline in 2020. The transmission rate dropped by 70% according to the state of emergency order (see “Background” for details about the state of emergency in Japan).

A computer script for the simulations is available at GitHub (https://github.com/yukifuruse1217/COVID_simulation_japan).

## RESULTS

**Figure 2:**
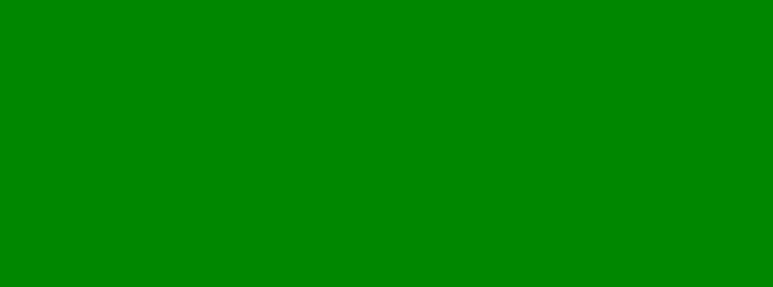
Cumulative numbers of COVID-19 deaths by simulation. Cumulative numbers of deaths by vaccination coverage, basic reproduction number, and vaccine efficacy in the simulation for 150 days are shown. **Panel A** is for very effective vaccines, and **Panel B** is for effective vaccines. The x-axis denotes the degree of transmission reduction due to nonpharmaceutical intervention restrictions. The y-axis is in a logarithmic scale.

Assuming the basic reproduction number of SARS-CoV-2 was 5.0, the vaccines were very effective, and the transmission was heterogeneous among age groups (**Table 1**), the cumulative number of COVID-19 deaths in one season (150 days) reached ~ 50 000 in the high vaccination coverage scenario when all NPI restrictions were lifted (**Figure 2**, Panel A). Without any restrictions, the death count surpassed 200 000 in the intermediate and low vaccination coverage scenarios. For comparison, the annual number of excess deaths due to influenza in Japan was approximately 10 000 [27]. If we sustained the modified lifestyle observed in 2020, which could reduce transmissions by 40% (Figure S1 in the **Online Supplementary Document**), the death count would be <10 000 in the high and intermediate vaccination coverage scenarios (**Figure 2**, Panel A).

**Figure 3:**
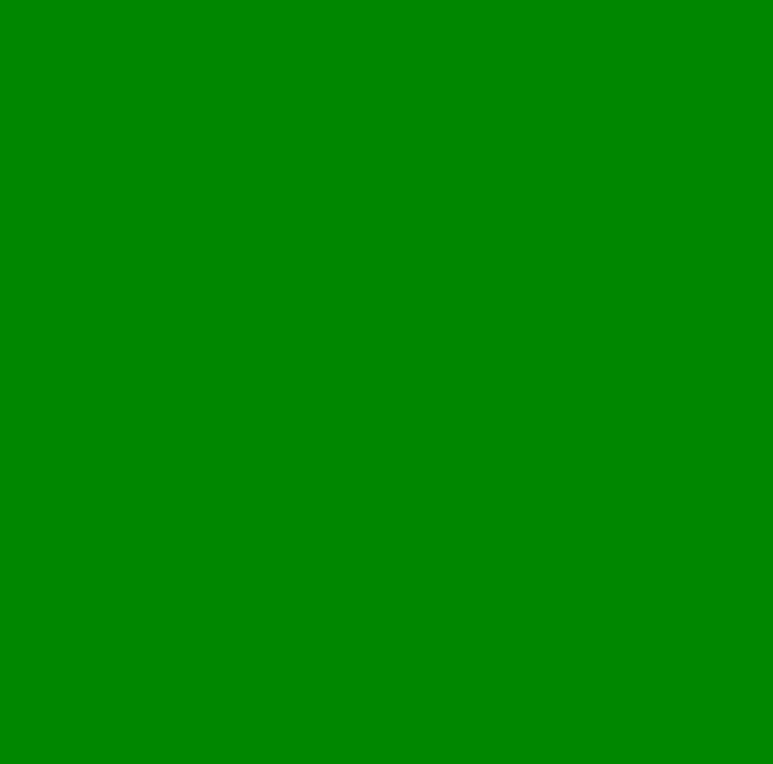
Cumulative numbers of COVID-19 infections and deaths by age group. Cumulative numbers of infections (**A**, **C**) and deaths (**B**, **D**) in the simulation for 150 days are shown by age group. The vaccination coverage (in three colors) and the vaccine efficacy (panels A and B for very effective vaccines; and panels C and D for effective vaccines) were subject to change. The basic reproduction number was set to 5.0. The x-axis denotes the degree of transmission reduction due to nonpharmaceutical intervention restrictions. The y-axis is in a linear scale.

Most infections occurred in children and young adults in all three vaccination coverage scenarios (**Figure 3**, Panels A and C). However, most deaths were observed among older adults (**Figure 3**, Panels B and D), even though we assumed that the vaccination coverage rate was especially high for the age group (**Table 1**).

If the basic reproduction number was 2.5, corresponding to the original strain, or 3.5, corresponding to the Alpha variant of SARS-CoV-2 (**Table 1**), we would be able to relax restrictions from the 2020 baseline (ie, 40%) even in the low vaccination coverage scenario (**Figure 2**, Panel A). On the other hand, we would keep requiring strict measures, such as those under a state of emergency, if the reproduction number of circulating viruses was 7.5 and the vaccination coverage was not high.

**Figure 4:**
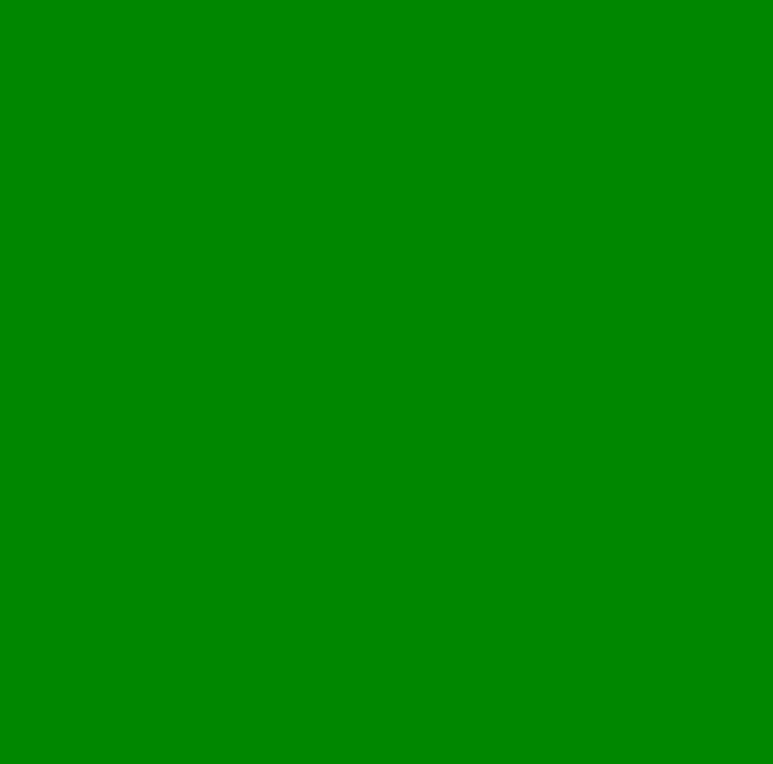
Cumulative numbers of COVID-19 infections and deaths by vaccination status. Cumulative numbers of infections (**A**, **C**) and deaths (**B**, **D**) in the simulation for 150 days are shown by vaccination status. The vaccination coverage (in three colors) and the vaccine efficacy (panels A and B for very effective vaccines; and panels C and D for effective vaccines) were subject to change. The basic reproduction number was set to 5.0. The x-axis denotes the degree of transmission reduction due to nonpharmaceutical intervention restrictions. The y-axis is in a linear scale.

**Figure 5:**
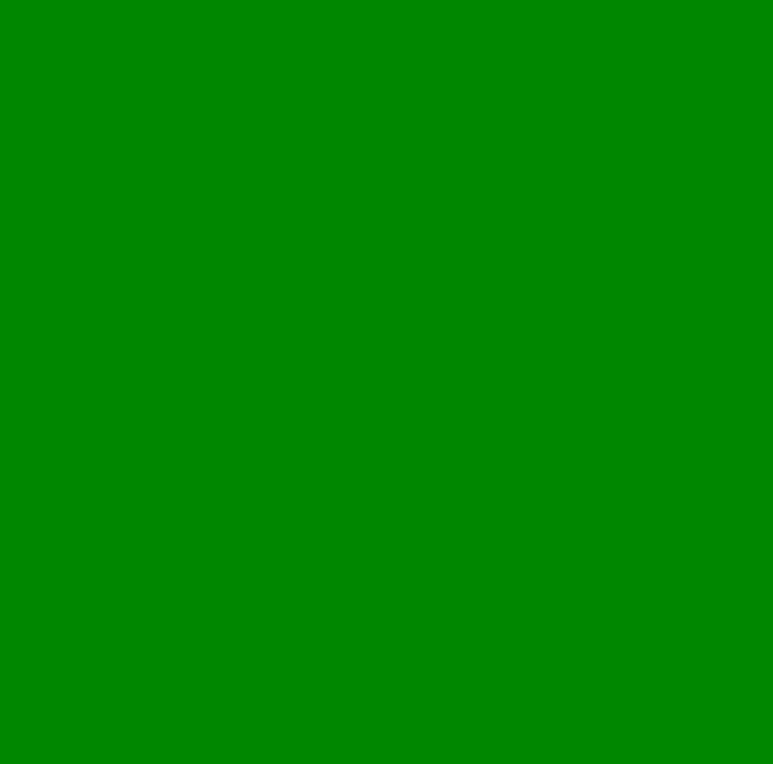
Incidence and mortality of COVID-19 per vaccination status. Cumulative numbers of infections (**A**, **C**) and deaths (**B**, **D**) in the simulation for 150 days were divided by the total number of either vaccinated or unvaccinated people to calculate incidence and mortality rates by vaccination status. The vaccination coverage (in three colors) and the vaccine efficacy (panels A and B for very effective vaccines; and panels C and D for effective vaccines) were subject to change. The basic reproduction number was set to 5.0. The x-axis denotes the degree of transmission reduction due to nonpharmaceutical intervention restrictions. The y-axis is in a linear scale.

As expected, the outcome would worsen if the vaccines were somewhat effective but not very effective (**Table 1**). With decreased vaccine efficacy for viruses with a basic reproduction number of 5.0, the cumulative number of COVID-19 deaths would be ~ 230 000 in the high vaccination coverage scenario if we lifted all NPI restrictions (**Figure 2**, Panel B). With very effective vaccines, most infections and deaths occurred in unvaccinated people (**Figure 4**, Panels A and B). In contrast, breakthrough infections among vaccinated people would account for a larger proportion of infected people and deaths in the high vaccination coverage scenario where the vaccines had decreased efficacy (**Figure 4**, Panels C and D). Still, incidence and mortality rates were consistently low for vaccinated people compared with unvaccinated people (**Figure 5**).

Suppose we achieved ~ 90% vaccination coverage for all age groups, as is the case for measles in many countries including Japan [28]. In that case, we could dramatically suppress the number of deaths due to COVID-19 (**Figure 1**, Panels A and B). The assumption of homogeneous viral transmission patterns increased the impact of the disease (Figure S2 in the **Online Supplementary Document**). The results of better control by herd immunity in a population with heterogeneous transmission patterns agree with the findings of a previous study [29].

We finally analyzed the temporal dynamics of future COVID-19 epidemic scenarios in Japan with the first assumption: the basic reproduction number of circulating viruses was 5.0, the vaccines were very effective, and the transmission was heterogeneous among age groups. We assumed that we would keep NPI restrictions as in 2020 (ie, 40% reduction in transmission) or halve the restrictions from 2020 (ie, 20% reduction in transmission) as a baseline. Moreover, we implemented a state of emergency, which can reduce transmissions by 70% (Figure S1 in the **Online Supplementary Document**), for 60 days when the number of patients with severe illness requiring mechanical ventilation, intensive care unit (ICU) admission, or extracorporeal membrane oxygenation surpassed 2000. In Japan, the highest number of such severe COVID-19 cases recorded by August 2021 was ~ 2000. This indicator corresponds to the burden on health systems such as the occupancy of ICU beds. We did not take seasonal effects, including meteorological factors and holiday seasons, into account in our simulation. Voluntary, temporary changes in people’s behavior without strict measures that affect transmission dynamics were also not considered.

**Figure 6:**
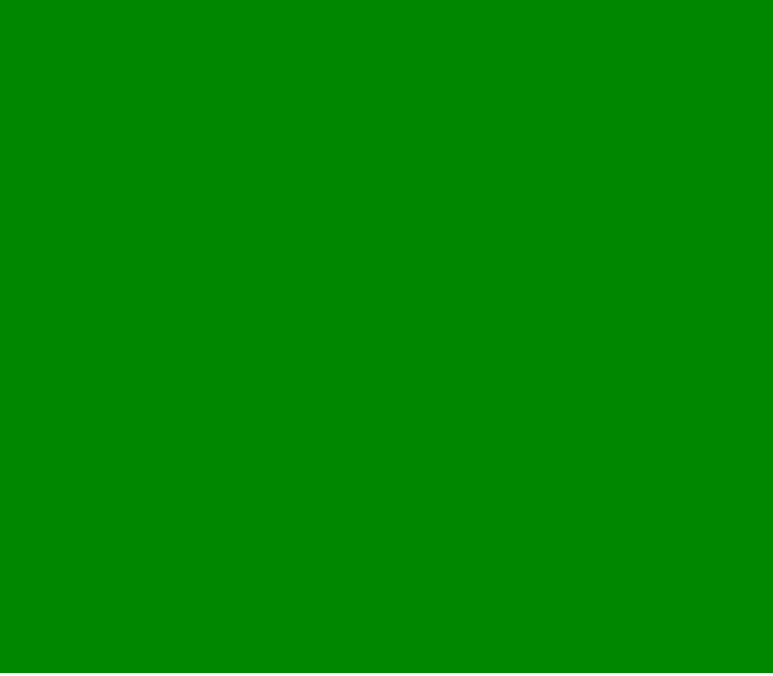
Temporal transmission dynamics of COVID-19 with a baseline transmission reduction and implementation of strict measures. The temporal dynamics of the numbers of newly infected people, severe cases, and cumulative deaths are shown. In the simulation, we assumed that transmission was reduced by 40% (**A**, **C**) or 20% (**B**, **D**) as baseline nonpharmaceutical intervention restrictions. The vaccination coverage (lines in three colors) and the vaccine efficacy (panels A and B for very effective vaccines; and panels C and D for effective vaccines) were subject to change. Strict measures reducing transmissions by 70% were implemented for 60 days when severe cases surpassed 2000.

An epidemic would not take off in the high vaccination coverage scenario with very effective vaccines and a 40% reduction in transmission as a baseline (**Figure 6**, Panel A). The intermediate vaccination coverage scenario required one-time implementation of strict measures. And, the low vaccination coverage scenario resulted in periodic implementation (several times in a year) of strict measures. If we relaxed our baseline restrictions from 40% to 20%, repetitive implementation of strict measures would be essential to control the epidemic in both the intermediate and low vaccination coverage scenarios (**Figure 6**, Panel B).

One-time implementation of strict measures was enough to keep the COVID-19 epidemic under control in the high vaccination coverage scenario with a 20% transmission reduction baseline with very effective vaccines (**Figure 6**, Panel B) and in the high vaccination coverage scenario with a 40% reduction baseline with vaccines of somewhat decreased efficacy (**Figure 6**, Panel C). However, relaxing restrictions to 20% would result in the occasional implementation of strict measures even in the high vaccination coverage scenario where vaccine efficacy was not optimal (**Figure 6**, Panel D).

## DISCUSSION

The results of our simulation study suggest that high vaccination coverage is important to control the COVID-19 epidemic. We can even lift or relax NPI restrictions when vaccination coverage and vaccine efficacy are high enough. However, this optimistic projection can be easily dashed when vaccination coverage is low, vaccine efficacy is insufficient, or circulating viruses are more transmissible than expected. A similar conclusion was reached by another simulation study based on situations in France [30]. Although we performed this study in the context of Japan, the results must have significant implications for other countries as well.

We did anticipate the need for periodic implementation of strict measures from the beginning of the COVID-19 pandemic [31-33]. However, we made the assumption when we did not yet have effective vaccines. With the development of effective vaccines, we hoped to eliminate the virus and return to normalcy (**Figure 2**, Panel A) [34,35]. The emergence of more transmissible variants and the possible reduction in vaccine efficacy have changed our perceptions of the disease.

The efficacy of vaccines could be even lower than our assumptions for the Delta and future variants [17,36-38]. Breakthrough infections among vaccinated people impose a serious concern as we have detected a substantial difference between very effective and effective vaccine scenarios in this study. We have to keep trying to increase vaccination coverage. It should be noted that the high proportion of vaccinated people in outbreaks does not mean vaccines are ineffective [39,40]. We showed that it could happen when vaccination coverage is high but the efficacy is not perfect but still good (Figures 4 and 5). We may have to administer booster shots and renew the components of vaccines to deal with immunity waning and keep up with emerging variants [41-43].

Although we did not explicitly consider the effect of waning immunity in our model, decreased vaccine efficacy against the Delta variant may have covered the point to some extent [20,44,45]. Since this study investigated the situation where all people who are willing to be vaccinated had been already fully vaccinated, we did not consider the time-varying, increasing proportion of vaccination coverage or partial protection by a single dose of vaccination in the simulation.

This study has several limitations. Most of the parameters used in this study were empirical ones obtained from data in Japan. However, there are several uncertainties and points we did not consider in the model. These include the difference in transmissibility from asymptomatic and symptomatic patients, the difference in vaccination efficacy among age groups, and the difference in the effectiveness of NPI restrictions among different populations. Because most of the reported epidemiological parameters such as the basic reproduction number of SARS-CoV-2 was calculated not separating asymptomatic and symptomatic patients [14-16], we regard combining them in a single compartment in our model as reasonable. Although the decreased vaccine efficacy in older adults was reported, its degree varies among studies [8,10,46]. It was difficult to set parameters to define the age group-specific vaccine efficacy in the present study. While the effect of NPI restrictions on people's mobility and contact pattern may differ by age group [47], it remains unclear how that would affect the transmission efficiency and the course of the epidemic. To develop better models, vigilant surveillance and further research are essential.

We assessed the impact of COVID-19 mainly by the number of deaths in this study. However, several remedies have already been developed, and others are currently under development [48,49]. They would reduce the disease’s fatality rate in the future. The burden on health systems was assessed by the number of severe cases in this study. Although the number of hospitalized patients might also be of interest for evaluating the burden, we could not take that into account. That was because the strategy about who to be hospitalized is different among prefectures in Japan. Almost all symptomatic cases including mild ones are hospitalized in some prefectures, while only patients requiring oxygen administration and patients with very high risk for developing severe illness are hospitalized in other prefectures. Furthermore, the effect of the sequela of COVID-19, dubbed “Long COVID,” and the economic impact of NPIs and their indirect effects on public health, including suicide, should be considered. The inclusion of stochasticity in the model is another way for further exploration in future studies.

## CONCLUSIONS

In conclusion, we should carefully explore a manageable degree of restrictions and their relaxation. The importance of the combination of vaccine rollout and NPIs has been shown in other studies as well [50,51]. We will have to keep bracing for occasional surges of the disease leading to strict measures, such as those under a state of emergency. Such strategies are essential even after most eligible people had been vaccinated. Although we assumed in our simulations that universal NPI restrictions would reduce transmissions, the use of vaccination certificates and negative test certificates may potentially help relax some restrictions. However, we need to address the relevant ethical issues involved [52].

## Additional material


Online Supplementary Document
