# Supplementary material for: Simulation of future COVID-19 epidemic by vaccination coverage scenarios in Japan
Source: J Glob Health. 2021 Nov 30;11:05025. doi: 10.7189/jogh.11.05025 (PMC8576362; doi:10.7189/jogh.11.05025)
Supplement: Online Supplementary Document [file jogh-11-05025-s001.pdf]

Online Supplementary Document

Table S1. Parameters and initial values for the compartmental model.

Age group (i)	Age (years)		
1	0–19		
2	20–39		
3	40–59		
4	≥60		
**Vaccination coverage scenario**	**Vaccination coverage (12–39 years, 40–59 years, ≥60 years)**	**Description**	**Source**
90% coverage	90%, 90%, 90%	Assuming 90% of eligible people got vaccinated	−
High coverage	75%, 80%, 90%	Corresponding to the sum of people who are willing to be vaccinated and those who cannot decide yet	(1)
Intermediate coverage	60%, 70%, 85%	Intermediate scenario between High and Low coverages	(1)
Low coverage	45%, 60%, 80%	Corresponding to a proportion of people who are willing to be vaccinated	(1)
No vaccination	0%, 0%, 0%	Scenario without vaccination	−
**Parameter**	**Description**	**Value**	**Source**
Basic reproduction number	Mean number of secondary cases produced by a single infector in a completely susceptible population	2.5, 3.5, 5.0, 7.5	(2–4)
$R_{i,j}$	Reproduction number from age group j to age group i (Maximal eigenvalue of the matrix $R_{i,j}$ corresponds to a basic reproduction number)	Supplementary Figure 2	[*1]
N1	Population of age group 1	23,000,000 (0–11 years, 130,000; 12–19 years, 100,000)*2	Census
N2	Population of age group 2	32,500,000	Census
N3	Population of age group 3	33,500,000	Census
N4	Population of age group 4	37,000,000	Census
$1/\gamma$	Mean generation time (days)	5	(5)
$1/\gamma + 1/\varepsilon$	Mean hospitalization period of severe cases (days)	19	[*1]
$\delta1$	Severity rate for age group 1	0.03%	[*3*4]
$\delta2$	Severity rate for age group 2	0.06%	(6)[*3]
$\delta3$	Severity rate for age group 3	1.0%	(6)[*3]
$\delta4$	Severity rate for age group 4	8.5%	(6)[*3]
$\delta1 \times \mu1$	Fatality rate for age group 1	0.005%	[*3*4]
$\delta2 \times \mu2$	Fatality rate for age group 2	0.01%	(6)[*3]
$\delta3 \times \mu3$	Fatality rate for age group 3	0.2%	(6)[*3]
$\delta4 \times \mu4$	Fatality rate for age group 4	5.7%	(6)[*3]
Ea	Vaccine efficacy to prevent infection	90% (very effective), 70% (effective)	(7–15)
1 − (1 − Ea) × Eb	Vaccine efficacy to prevent severe illness and death	95% (very effective), 90% (effective)	(7–16)
1 – Ec	Vaccine efficacy to reduce infectivity from patients with a breakthrough infection	50% (very effective), 25% (effective)	(17,18)[*5]
**Compartment**	**Description**	**Initial value**	
Sni	Unvaccinated people in age group i	Ni × 0.99 × (1 − vaccination coverage for age group i)	
Svi	People vaccinated but not protected in age group i	Ni × 0.99 × (vaccination coverage for age group i) × (1 − Ea)	
Vi	Pople protected by vaccination in age group i	Ni × 0.99 × (vaccination coverage for age group i) × Ea	
Ini	Infected (infectious) people among those unvaccinated in age group i	100	
Ivi	Infected (infectious) people among those vaccinated in age group i	0	
Ci	Infected people in age group i with severe illness requiring mechanical ventilation, intensive care unit admission, or extracorporeal membrane oxygenation	0	
Ri	Recovered people in age group i (assuming 1% of the total population had been already infected in Japan)	Ni × 0.01	
Di	Dead people due to COVID-19 in age group i	0	

[*1] Determined using empirical data from Japan.

[*2] In age group 1, only those aged 12–19 years are eligible for vaccination.

[*3] Data are from 2020. The change in pathogenicity by the Delta variant is probable (19,20), but that was not considered in the simulation due to the lack of data in Japan.

[*4] Due to the small number of COVID-19 cases for age group 1 in Japan, we could not estimate the severity and fatality rates for the group. We assumed the rates to be half of those for age group 2.

[*5] Although the vaccine efficacy to reduce infectivity from patients with a breakthrough infection for the Delta variant might decline (21,22), the efficacy seems still maintained to some degree (23,24). We assumed the efficacy to be half of that for the very effective scenario.

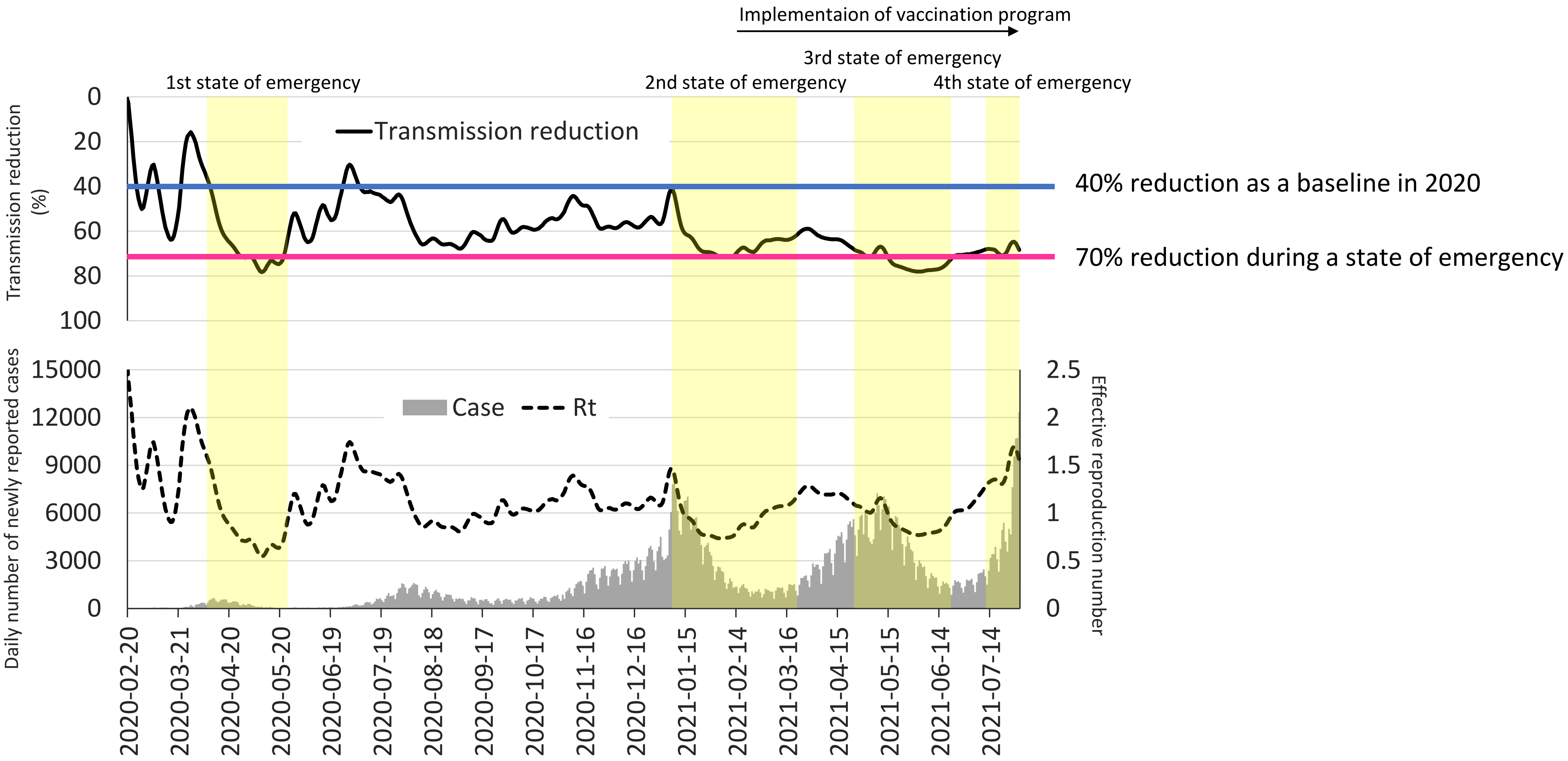

Figure S1. Transmission dynamics of COVID-19 and transmission reduction in Japan from 20 February 2020 to 31 July 2021.

The daily numbers of confirmed COVID-19 cases were retrieved from the website of the Ministry of Health, Labour and Welfare. The effective reproduction number (Rt) was calculated using EpiEstim with the parameter of a serial interval set to 5 days (5,25). Reduction in transmission was calculated as follows: (1 – Effective reproduction number / Basic reproduction number). The basic reproduction was set to 2.5 (original strain), 3.5 (Alpha variant), or 5.0 (Delta variant) (2–4). The transitions from the original strain to the Alpha variant and from the Alpha variant to the Delta variant were considered to have occurred in February–April 2021 and July 2021, respectively (26). Shaded areas in yellow indicate periods under a state of emergency. Note that transmission reduction was achieved by nonpharmaceutical interventions plus the effect of vaccination after February 2021.

A

Homogeneous transmission

≥60Y (age group 4)	1	1	1	1
40–59Y (age group 2)	1	1	1	1
20–39Y (age group 2)	1	1	1	1
0–19Y (age group 1)	1	1	1	1
To (i) / From (j)	0–19Y (age group 1)	20–39Y (age group 2)	40–59Y (age group 3)	≥60Y (age group 4)

Heterogeneous transmission

≥60Y (age group 4)	1	4	4	4
40–59Y (age group 2)	1	4	16	1
20–39Y (age group 2)	1	16	4	1
0–19Y (age group 1)	4	4	1	1
To (i) / From (j)	0–19Y (age group 1)	20–39Y (age group 2)	40–59Y (age group 3)	≥60Y (age group 4)

B **Homogeneous transmission with very effective vaccines**

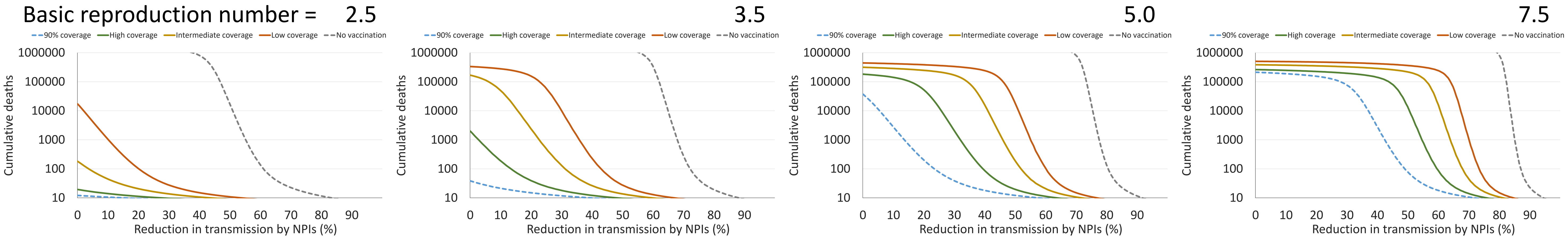

C **Homogeneous transmission with effective vaccines**

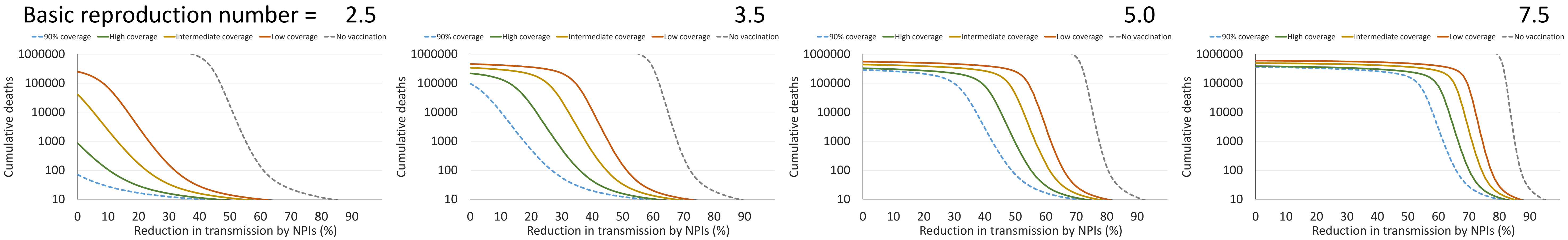

Figure S2. Cumulative numbers of COVID-19 deaths by simulation with a homogeneous model.

(A) Relative values of reproduction numbers from age group *j* to age group *i* are shown for the homogeneous and heterogeneous models. In the heterogeneous model, we assumed that transmissions from people aged 20–59 years and transmissions within the same age group were four times higher than the other transmission pairs. Four-fold higher transmissions within the same age group were observed in empirical data from Japan. Four-fold higher transmissions

from those aged 20–59 years were determined in this study as we validated that the coefficient of 4 in our simulation fitted well with the observation data that ~70% of all infected people in Japan were aged 20–59 years before the implementation of the vaccination program. The numbers in the matrices are relative values, and they were proportionally adjusted so that the maximal eigenvalue of the matrix corresponds to a basic reproduction number. (B, C) Cumulative numbers of deaths by vaccination coverage, basic reproduction number, and vaccine efficacy in the simulation for 150 days, assuming a population with homogeneous transmission patterns, are shown. Panel B is for very effective vaccines, and Panel C is for effective vaccines. The x-axis denotes the degree of transmission reduction due to nonpharmaceutical intervention restrictions. The y-axis is in a logarithmic scale. Results for the heterogeneous model are shown in Figure 2.

Supplementary references

1. BuzzFeed. Sixty percent of people are willing to get vaccinated in Japan [in Japanese]. 2021. Available from: https://www.buzzfeed.com/jp/naokoiwanaga/covid-19-vaccine-chousa. Accessed: 12 August 2021.
2. Li Q, Guan X, Wu P, Wang X, Zhou L, Tong Y, et al. Early Transmission Dynamics in Wuhan, China, of Novel Coronavirus–Infected Pneumonia. N Engl J Med. 2020;382:1199–207.
3. Wu JT, Leung K, Leung GM. Nowcasting and forecasting the potential domestic and international spread of the 2019-nCoV outbreak originating in Wuhan, China: a modelling study. Lancet. 2020;395:689–97.
4. Campbell F, Archer B, Laurenson-Schafer H, Jinnai Y, Konings F, Batra N, et al. Increased transmissibility and global spread of SARS-CoV-2 variants of concern as at June 2021. Eurosurv. 2021;26:2100509.
5. Rai B, Shukla A, Dwivedi LK. Estimates of serial interval for COVID-19: A systematic review and meta-analysis. Clin Epidemiol Glob Heal. 2021;9:157–61.
6. Ministry of Health Labour and Welfare (Japan). Eleven facts about COVID-19 [in Japanese]. 2021. Available from: https://www.mhlw.go.jp/content/000788485.pdf. Accessed: 12 August 2021.
7. Angel Y, Spitzer A, Henig O, Saiag E, Sprecher E, Padova H, et al. Association Between Vaccination With BNT162b2 and Incidence of Symptomatic and Asymptomatic SARS-CoV-2 Infections Among Health Care Workers. JAMA. 2021;325:2457–65.
8. Dagan N, Barda N, Kepten E, Miron O, Perchik S, Katz MA, et al. BNT162b2 mRNA Covid-19 Vaccine in a Nationwide Mass Vaccination Setting. N Engl J Med. 2021;384:1412–23.
9. Tang L, Hijano DR, Gaur AH, Geiger TL, Neufeld EJ, Hoffman JM, et al. Asymptomatic and Symptomatic SARS-CoV-2 Infections After BNT162b2 Vaccination in a Routinely Screened Workforce. JAMA. 2021;325:2500–2.
10. Baden LR, Sahly HM El, Essink B, Kotloff K, Frey S, Novak R, et al. Efficacy and Safety of the mRNA-1273 SARS-CoV-2 Vaccine. N Engl J Med. 2020;384:403–16.
11. Tenforde MW. Effectiveness of Pfizer-BioNTech and Moderna Vaccines Against COVID-19 Among Hospitalized Adults Aged ≥65 Years — United States, January–March 2021. MMWR Morb Mortal Wkly Rep. 2021;70:674–9.
12. Ministry of Health (Israel). Explanation About the Effectiveness of the Vaccine for Coronavirus in Israel. 2021. Available from: https://www.gov.il/en/departments/news/06072021-04. Accessed: 12 August 2021.
13. Riley S, Eales O, Haw D, Wang H, Walters CE, Ainslie KEC, et al. REACT-1 round 13 interim report: acceleration of SARS-CoV-2 Delta epidemic in the community in England during late June and early July 2021. medRxiv. 2021;2021.07.08.21260185.
14. Puranik A, Lenehan PJ, Silvert E, Niesen MJM, Corchado-Garcia J, O'Horo JC, et al. Comparison of two highly-effective mRNA vaccines for COVID-19 during periods of Alpha and Delta variant prevalence. medRxiv. 2021;2021.08.06.21261707.
15. Bernal JL, Andrews N, Gower C, Gallagher E, Simmons R, Thelwall S, et al. Effectiveness of Covid-19 Vaccines against the B.1.617.2 (Delta) Variant. N Engl J Med. 2021;385:585-594.
16. Polack FP, Thomas SJ, Kitchin N, Absalon J, Gurtman A, Lockhart S, et al. Safety and Efficacy of the BNT162b2 mRNA Covid-19 Vaccine. N Engl J Med. 2020;383:2603–15.
17. Harris RJ, Hall JA, Zaidi A, Andrews NJ, Dunbar JK, Dabrera G. Effect of Vaccination on Household Transmission of SARS-CoV-2 in England. N Engl J Med. 2021;385:759-760.
18. Shah ASV, Gribben C, Bishop J, Hanlon P, Caldwell D, Wood R, et al. Effect of Vaccination on Transmission of SARS-CoV-2. N Engl J Med. 2021;NEJMc2106757.
19. Sheikh A, McMenamin J, Taylor B, Robertson C. SARS-CoV-2 Delta VOC in Scotland: demographics, risk of hospital admission, and vaccine effectiveness. Lancet. 2021;397:2461–2.
20. Twohig KA, Nyberg T, Zaidi A, Thelwall S, Sinnathamby MA, Aliabadi S, et al. Hospital admission and emergency care attendance risk for SARS-

CoV-2 delta (B.1.617.2) compared with alpha (B.1.1.7) variants of concern: a cohort study. Lancet Infect Dis. 2021;S1473-3099(21)00475-8.

21. Musser JM, Christensen PA, Olsen RJ, Long SW, Subedi S, Davis JJ, et al. Delta variants of SARS-CoV-2 cause significantly increased vaccine breakthrough COVID-19 cases in Houston, Texas. medRxiv. 2021;2021.07.19.21260808.

22. Riemersma KK, Grogan BE, Kita-Yarbro A, Halfmann P, Kocharian A, Florek KR, et al. Shedding of Infectious SARS-CoV-2 Despite Vaccination when the Delta Variant is Prevalent - Wisconsin, July 2021. medRxiv. 2021;2021.07.31.21261387.

23. Shamier MC, Tostmann A, Bogers S, Wilde J De, IJpelaar J, Kleij W Van Der, et al. Virological characteristics of SARS-CoV-2 vaccine breakthrough infections in health care workers. medRxiv. 2021;2021.08.20.21262158.

24. Eyre DW, Taylor D, Purver M, Chapman D, Fowler T, Pouwels KB, et al. The impact of SARS-CoV-2 vaccination on Alpha & Delta variant transmission. medRxiv. 2021;2021.09.28.21264260.

25. Cori A, Ferguson NM, Fraser C, Cauchemez S. A new framework and software to estimate time-varying reproduction numbers during epidemics. Am J Epidemiol. 2013;178:1505–12.

26. Ito K, Piantham C, Nishiura H. Predicted dominance of variant Delta of SARS-CoV-2 before Tokyo Olympic Games, Japan, July 2021. Eurosurv. 2021;26:2100570.
